# Vitamin D Receptor Agonists: Suitable Candidates as Novel Therapeutic Options in Autoimmune Inflammatory Myopathy

**DOI:** 10.1155/2014/949730

**Published:** 2014-05-07

**Authors:** Clara Crescioli

**Affiliations:** Section of Health Sciences, Department of Movement, Human and Health Sciences, University of Rome Foro Italico, 00135 Rome, Italy

## Abstract

The primary aim in the treatment of autoimmune inflammatory myopathies (IMs) is to recover muscle function. The presence of immune/inflammatory cell infiltrates within muscle tissues represents the common feature of different IM subtypes, albeit a correlation between muscular damage extent and inflammation degree is often lacking. Treatments for IMs are based on life-long immunosuppressive therapy, with the well known adverse effects; recovery is incomplete for many patients. More effective therapies, with reduced side-effects, are highly desirable. Vitamin D receptor (VDR) agonists emerge to retain pleiotropic anti-inflammatory properties, since they regulate innate and adaptive immunity by switching the immune response from proinflammatory T helper 1 (Th1) type to tolerogenic T helper 2 (Th2) type dominance. In skeletal muscle cells less hypercalcemic VDR ligands target powerful mediators of inflammation, such as TNF**α** and TNF**α** driven paths, without affecting immune or muscle cells viability, retaining the potentiality to counteract Th1 driven overreactivity established by the self-enhancing inflammatory loop between immune and skeletal muscle cells. This review summarizes those features of VDR agonists as candidates in future treatment of IM.

## 1. Introduction

Increasing evidence points out that vitamin D, beside bone metabolism and calcium homeostasis regulation, plays a pivotal role in maintaining the functionality of many other tissues, including skeletal muscle. A direct association of vitamin D status with skeletal muscle fiber composition, muscle power and force, or physical performance has been documented by several studies in old or young human population [1, 2]; remarkably, vitamin D supplementation is associated with improvements in muscle performance and fall reduction [1, 3–6].

Experimental models of VDR null mutant mice document diffused muscle fiber abnormalities and severe alterations in muscle cell differentiation or fiber development/maturation [7–9]; in humans, VDR gene polymorphisms have been associated with muscle strength defects, as recently reported [1, 10]. Direct effects of vitamin D on muscle cell proliferation, differentiation, and myotube size have been recently proposed in a murine experimental* in vitro* model [11]. Skeletal muscle is a well known target tissue of vitamin D action and the association between severe vitamin D deficiency and myopathy has been recognized since and recently confirmed [1, 12]. Myopathy is characterized by severe myofiber degeneration and muscle wasting; in particular, IMs are a wide range of autoimmune diseases, collectively known as myositis, characterized clinically by reduced muscle endurance and weakness, chronic inflammation, and infiltration by immune/inflammatory cells in skeletal muscles. Since both adaptive and innate immunity are involved in IMs, the mainstay treatment is directed to suppress or modify immune cell activity and is based on high dose corticosteroid combined with immunosuppressive drugs, as steroid-sparing agents [13–15]. However, most of IM patients have just a partial clinical improvement, few recover muscle performance, and about 25% are refractory to those drugs and left with disability [13–15], suggesting that pharmacological targeting the immune system may be not enough for satisfactory therapeutic effects.

Much more interest has been recently addressed to the muscular component, as an active counterpart dialoguing with the immune system during inflammation throughout the production of cytokines and chemokines, highly chemotactic peptides. In this light, skeletal muscle cells, behaving as immunoactive structures, could be also hypothesized to be therapeutic targets as well.

Advances in clinical and bench research highlight the vitamin D impact on muscle function and morphology, either in physiologic or pathologic conditions [16–18]; also, VDR agonists emerge to exert pleiotropic activities in (auto)immune regulation by targeting both immune and resident cells [19–21].

This review aims to offer an overview on VDR agonists as potential novel therapeutic tools to control inflammation in IMs; in particular, biomolecular pathway(s) and inflammatory mediators within skeletal muscle cells engaged in IM pathogenesis, such as the cytokine TNF*α* and the chemokine CXCL10, will be discussed as intracellular pharmacological target(s) of nonhypercalcemic VDR agonists.

## 2. Pathogenic Mechanisms of IMs

IMs are a heterogeneous group of systemic autoimmune diseases subclassified in distinct subgroups, that is, idiopathic dermatomyositis (DM), polymyositis (PM), inclusion body myositis (IBM), the most studied ones, necrotizing autoimmune myositis, and myositis associated with systemic disorders on the basis of some clinical and histological differences [22–24]. Muscle weakness, fatigue, and elevated serum muscle enzymes, together with myofiber degeneration/fibrosis and mononuclear cell infiltration represent, respectively, clinical and histological features common to all subtypes.

Different pathogenic mechanisms have been hypothesized due to distinct predominating localization/phenotype of the inflammatory infiltrates, that is, while a striking dominance of CD4^+^ T cells has been reported at perivascular/perimysial sites, as often found in DM, endomysial infiltrates are dominated by CD8^+^ T cells, as more frequently observed in PM and IBM; the presence of B lymphocytes, which seem to preferentially target the microvascular component in DM, is considered less critical in PM [25]. Those differences, however, appear to be an oversimplification of the reality: an overlap between clinical phenotypes, immunotypes, and histopathology has been often depicted and frequently mirrors an overlap in diagnostic criteria as well [15, 25–27]. The inflammatory molecules and mediators involved in muscles affected by myositis are highly similar, given that some essential molecular paths engaged in immune response (innate and adaptive) are shared between the different IM subsets. The presence of autoantibodies, frequently detected in PM and DM, autoreactive lymphocytes, together with overexpression of major histocompatibility complex (MHC, or HLA, human leucocyte antigen) molecules on the surface of the affected myofibers albeit, at different degree, represent common traits of immune-mediated diseases. HLA molecules mediate the immune response by presenting processed antigen peptides, either self- or not-self, to activated T cells. In particular, T cells with Th1 immune reaction predominance, macrophages, and dendritic cells (DC) are found in muscles of the different subgroups of IMs [25, 28]. The presence of B lymphocytes, natural killer (NK) cells, or antigen presenting cell (APC) other than DC—such as endothelial or skeletal muscle cells themselves—have been also observed in all types of IMs [29–31]. However, albeit pathogenic mechanisms still are unclear, Th1 cells and macrophages play a pivotal role in IM pathogenesis: their local accumulation likely contributes to the deposition of immune complexes within skeletal muscles [32, 33] through the release of functional molecules, such as cytokines and chemokines; once present within the muscular microenvironment, they are able to cause damages directly to fibers and capillaries of the affected muscles [34]. The expression of cytokines as IFN*γ*, TNF*α*, and several interleukins (IL), such as IL-6, IL-4, IL-17, or IL-12p40, is increased in muscle biopsies of IM subjects [35–38]. Interactions between cytokines/chemokines and lymphocytes, suggested to be the link between innate and adaptive immunity, are likely critical for IM type and stage [34]; in addition intrinsic mechanisms in skeletal muscle appear to be highly significant in IM pathogenesis as well.

## 3. Skeletal Muscle as an Immunoactive Organ

The immunocompetence of nonimmune tissues has been recognized as determinant to drive the course of immune-inflammatory processes in several diseases, from organ rejection to autoimmune pathologies [39, 40]. Skeletal muscle cells are nowadays considered not only targets of immunological injury but actual active structures with intrinsic immunological capabilities [41–43].

### 3.1. Chemokines and Cytokines in Skeletal Muscle

Skeletal muscle is now considered as a secretory organ able to produce and release some cytokines—also termed myokines within the specific tissue context—to communicate with other organs, either in physiological conditions, that is, under contraction [42], or pathological conditions, as in inflammatory processes [43].

In particular, chemokines, a class of small cytokines with potent chemotactic activity, such as IL-8 (CXCL8), Mig (CXCL9), IP-10 (CXCL10), RANTES (CCL5), and MCP-1 (CCL2), are overexpressed during myositis in infiltrating inflammatory cells, extracellular matrix, and muscle fibers [32, 44–47]. Those molecules seem to have relevance for the immune pathogenesis of IMs because they promote and facilitate activated Th1 type cell trafficking to muscle tissues.

In line with this view, we have previously confirmed the importance of TNF*α* in IMs, suggesting new molecular insight(s) involving the important role of the chemokine CXCL10 in muscular inflammation [48, 49].

### 3.2. CXCL10 in Skeletal Muscle Inflammation

CXCL10 (or IP-10 10 kD IFN*γ*-induced protein) is a small peptide of CXC chemokine subfamily known to modulate innate and adaptive immune responses by controlling leukocyte trafficking [40, 50]. It is secreted by several types of immune and resident cells under proinflammatory conditions [40]. It is known to polarize T cells towards Th1 type dominance and seems to be directly associated to disease pathogenesis: through local tissue accumulation, it triggers and perpetuates a self-promoting inflammatory loop by interacting with its specific receptor CXCR3 on T, NK, B cells, macrophages, and DCs [40, 51–54]. Notably, human skeletal muscle cells challenged by inflammatory stimuli secrete significant amount of CXCL10 (virtually absent in basal condition), likely throughout a TNF*α*-driven mechanism: TNF*α*/TNF*α* receptor (TNF*α*R) system seems the critical one in promoting muscular inflammation at cellular level in human skeletal muscle cells, involving nuclear transcription factor *κ*B (NF-*κ*B), C-Jun NH2-terminal kinase (JNK), and extracellular signal-regulated kinase (ERK) intracellular path activation [48, 49]. In particular, the specific blockage of NF-*κ*B and JNK signaling significantly reduced CXCL10 secretion [49]. Thus, albeit CXCL10 is by definition an IFN*γ*-induced chemokine, it seems to be driven almost exclusively by TNF*α* in inflammatory processes within human skeletal muscle cells. That is, in our opinion, not so surprising.

### 3.3. The Route of TNF*α*

TNF*α* is known as one of the essential cytokines in promoting muscular inflammation at cellular level [55, 56]; it has been recently confirmed to be a key mediator involved in IM pathogenesis and, consequently, emerges as a potential therapeutic target [57–59]. Accordingly, the neutralization of TNF*α* activity with specific antibodies, TNF*α*R antagonists, or NF-*κ*B inhibitors has been investigated in experimental animal models of myositis,* in vitro* and* in vivo* [60, 61]. Of notice, TNF*α* pharmacological blockade by the neutralizing antibody infliximab or the soluble TNF*α*R etanercept, already used in clinics for the treatment of rheumatoid arthritis or Crohn's disease, has been extended to IMs, although with some caution [62–65]. Thus far, as from our and other's studies, targeting TNF*α*, TNF*α*R II—the subtype mainly engaged in immune response regulation [48, 66, 67]—and TNF*α*-related pathways—directly associated with CXCL10 production by skeletal muscle cells [49]—might be beneficial for IM treatment.

Hence, the capability of some VDR agonists to target TNF*α*, as shown by different studies, seems quite intriguing, and we would like to point out VDR agonist skill to selectively impair TNF*α* signaling during inflammation processes in human skeletal muscle cells.

## 4. VDR Agonists as Protolerogenic Molecules

It is known that vitamin D plays a role in the control of immune cell function through VDR, with important effects onto the immune-mediated response toward protolerogenic dominance [68–72]. VDR agonists are able to attenuate excessive Th1-driven inflammation and avoid downstream Th1 polarization during inflammatory processes involved in allo- or autoimmune response, that is, in organ transplant rejection or autoimmune diseases, as recently addressed in a review on the topic [39].

Herein, we would like to underline that the protolerogenic activity of VDR relies on their capability to control maturation, differentiation, and activation of different type of immune cells, that is, monocytes, macrophages, B and T lymphocytes, neutrophils, and DC, throughout the activation of VDR, either constitutively present or induced in the majority of the immune cells [73, 74].

Since the pioneering studies by Bhalla et al. [75], VDR agonists have been documented to inhibit selectively Th1 cell development [76, 77] and directly Th1-type cytokines, such as IL-2 and IFN*γ* [78–80]. In particular, while IL-2 inhibition is linked to an impairment of the transcription factor nuclear factor of activated T cells (NF-AT) complex formation, VDR ligand-induced IFN*γ* negative regulation has been explained by a direct interaction of the ligand-bound VDR complex with vitamin D responsive element (VDRE) within IFN*γ* promoter [78–80]. However, some controversy arises against direct effects of VDR ligands onto IFN*γ* inhibition [81]. Furthermore, T-cell activity can be inhibited by a VDR-mediated indirect mechanism through the downregulation of the expression of the MHC class II molecules and CD40, CD80, and CD86 costimulatory proteins in DC. DC are, indeed, well known targets of VDR ligands, which markedly impair IL-12—by targeting NF-*κ*B, through Rel-B and c-Rel NF*κ*B-related protein [82, 83]—and increase IL-10 production [81, 84–86]. The prevention of DC differentiation and maturation, activation, and survival leads to DC protolerogenic phenotype and function along with T-cell hyporesponsiveness, as shown by* in vivo* and* in vitro* studies [87, 88]. By the induction of protolerogenic DC phenotype, VDR ligands seem responsible for CD4^+^CD25^+^ regulatory T-cell enhancement [89, 90].

In addition, it is quite clear that VDR ligands directly target also Th17 cell subtype, as shown by the reduction of Th17 cytokines, such as IL-17A, IL-17F, and IL-22 by memory T cells in patients with early rheumatoid arthritis (RA) [91].

An enhanced development of Th2 type cells by VDR agonists throughout a direct effect on naïve CD4^+^ cells has been reported [92]. A direct enhancement of Th2 type genes (i.e., IL-10, IL-4) is favored by VDR agonists while gene transcription of Th1/Th17 type cytokines (i.e., IL-2, IL-6, IL-12, IL-17, and IL-23) is declined [93, 94]. Therefore, a definite switch of Th1 cell response towards Th2-mediated events occurs. Moreover, since macrophages [91, 95, 96], DC [97], and T cells [91] produce 1,25(OH)_2_D_3_, a contribution of this hormone to physiologically regulate innate and adaptive immunity could be speculated.

B cells, due to CYP27b1 (1alpha-hydroxylase) expression, have been hypothesized to be capable of autocrine/intracrine synthesis/response to vitamin D as well [98, 99]. In particular, vitamin D seems to predominantly modulate human naïve B cells activation through the VDR target gene cyp24a1 and NF-*κ*B [99] regulation.

**Figure 1:**
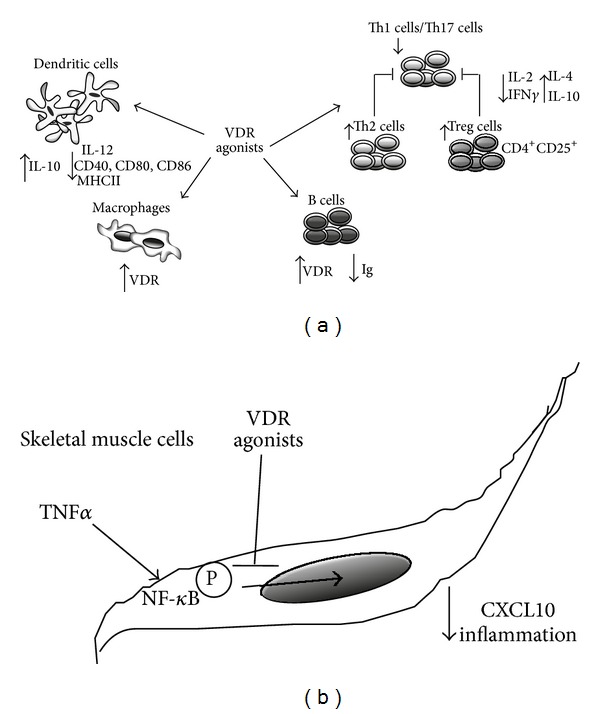
Schematic representation of protolerogenic effects of VDR ligands onto (a) immune cells, such as Th1/Th17, Th2, Treg subsets, DC, and macrophages and (b) skeletal muscle cells. Common targets, such as NF-*κ*B, are involved to attenuate inflammation and promote a shift towards Th2 protolerogenic subtype dominance.

So far, the anti-inflammatory feature of VDR agonists exerted onto several types of immune cells depends not only on VDR expression, but, and maybe especially, on the presence of common targets in their signal transduction pathways, such as the NF-*κ*B, downstream of TNF*α* [83, 100]. NF-*κ*B is a key mediator of cytokine/chemokine-induced inflammation in several types of tissue resident cells and, notably a VDR agonist tissue target as well [48, 101–103] (Figure 1).

## 5. VDR Agonists as Therapeutic Tools in IMs

Based on the capacity to counteract NF-kB activation also in resident cells, VDR agonists cause a significant reduction in local release of potent chemotactic factors—cytokines and chemokines—that, in turn, reduces the recruitment of immune cells (Th1 cells, macrophages, and DC) to the site of inflammation [18, 19, 80, 83]. As a result, the mechanisms underlying the self-enhancing inflammatory loop between immune and resident cells are likely impaired. So far, the feature of VDR agonists to counteract the path downstream of TNF*α* appears particularly relevant in view of a break up during inflammatory processes. Indeed, by interfering with NF-*κ*B nuclear translocation VDR agonists attenuate inflammation in several organ cell types such as adipocytes, thyrocytes, cardiomyocytes, in association to a decrease in proinflammatory cytokine production [20, 21, 104, 105].

We have recently reported on the effect of less-hypercalcemic VDR agonist BXL-01-0029 in human skeletal muscle cells under maximal inflammatory stimuli; it decreases CXCL10 secretion with the highest potency versus other current immunosuppressants and specifically deactivates TNF*α* pathways: JNK phosphorylation is reduced and, quite remarkably in our opinion, NF-*κ*B activation is prevented, while Stat1 activation, downstream of IFN*γ*, is unaffected. Hence, it appears that BXL-01-0029-induced inhibition of TNF*α* signal path may be sufficient to significantly decrease CXCL10 secretion by human skeletal muscle cells. This result seems quite intriguing considering the pivotal role of TNF*α* in muscular inflammation. Accordingly, in human skeletal muscle cells, differently from other cell types, such as human cardiomyocytes, thyrocytes, and renal cells, CXCL10 secretion seems to be essentially dependent on TNF*α*-driven mechanisms.

As previously addressed, counteracting TNF*α* signal and, therefore, CXCL10 local accumulation might result in a relevant crumble in the self-enhancing inflammatory loop established between immune infiltrating cells and resident muscular cells. This effect appears even more significant when considering the limited efficacy of the current immunosuppressants in controlling inflammation in IMs.

An additional consequence of TNF*α* detrimental effects is a possible dysregulation of mitochondrial metabolic pathways occurring in inflammatory muscle diseases [106, 107]: abnormalities in energy regulating paths and deficiencies in glycolytic enzymes have been often observed in IM fibers with more pronounced damages [108, 109]. Even though those mechanisms have still to be fully clarified, myoblasts, immature myofibers, and proinflammatory cytokines, such as TNF*α* and IL-15, seem the focal point for a cross talk between muscle inflammation and metabolism [34]. So far, targeting proinflammatory muscular cytokines secretion directly in myoblasts may be a quite helpful therapeutic strategy, considering the different possible beneficial effects.

The potential therapeutic application of BXL-01-0029 has been previously shown in nonobese diabetic (NOD) mice, who develop a pathogenesis similar to the human autoimmune type 1 diabetes (T1D), where VDR agonist-induced block of NF-*κ*B nuclear translocation in pancreatic islets is associated to a significant decrease both in Th1 cell organ infiltration and CXCL10 secretion [110]. We have also previously reported that BXL-01-0029 could be a potential steroid-sparing agent in the current immunosuppressant cocktails used to control inflammation in heart or renal rejection after transplantation [21, 101]. Notably, BXL-01-0029 similarly to elocalcitol, another nonhypercalcemic VDR agonist, does not affect cell viability in several types of organ resident cells and in CD4^+^ T cells, while significantly decreasing Th1- and Th17-cytokine secretion [20, 101]. Conversely, the majority of immunosuppressants have been designed to reduce the number of immune cells; this specific effect accomplishes the appearance of the well known noxious side effects of immunosuppressive drugs, that is, from metabolic disturbances, to opportunistic infections or tumor development [13–15]. So far, VDR agonists are likely able to control immune reaction acting essentially onto the production of mediators of inflammation, cytokines, and chemokines.

It cannot be ignored that circulating cytokine level are related also to the disease stage, such as acute or chronic, reflecting Th1 or Th2 dominance; in this regard, and in line with a previous study in IM patients in active phase of the diseases [111], we have recently reported that, in sera of subjects at time of diagnosis with IMs CXCL10 is higher than in matched controls, and, importantly, is the highest as compared to some other circulating Th1 cytokines—such as TNF*α*, IFN*γ*, IL-8, IL-6, MCP-1, MIP-1*β* (Th1 type), and IL-10 (Th2 type) [49]. Indeed, CXCL10, as Th1 type chemokine, participates to the early events in inflammatory/immune response and, even more important, is thought to trigger the reaction next to the antigenic challenge [54]. So far, we speculate that pharmacological targeting systemic and especially local muscular CXCL10 production with VDR agonists could result a particularly advantageous approach from the early stage of myositis.

## 6. Remarks and Conclusion

VDR agonists exert overall repressive effects onto Th1 polarized immune response, which dominates in inflammation, toward a more regulatory Th2 phenotype molecules, which dominate in tolerogenicity. Albeit the current controversy regarding VDR expression in adult skeletal muscle [112], as from our and other data, it appears that VDR agonists exert rapid anti-inflammatory effects directly in skeletal muscle cells. Whether VDR is expressed in skeletal muscle is still a debated issue [1, 112, 113]; the lower basal level in muscle as compared to duodenal cells—widely used as positive control—has been suggested as a possible cause of missing VDR detection in muscle cells [114]. Studies onto contractility and myogenesis showed that VDR is present and engaged in rapid nongenomic vitamin D-induced activation of tyrosine phosphorylation cascade in muscle cells [115]. Several lines of evidence indicate a membrane-associated VDR as the mediator of vitamin D-induced rapid events and, recently, the classical VDR located in caveolae has been shown to mediate vitamin D fast nongenomic signaling in skeletal muscle [114]. The existence of another cell surface receptor for vitamin D, named membrane associated, rapid response steroid binding (MARRS) has been reported in muscle [116].

Albeit the question on whether VDR exists in fully differentiated muscle or plays its pivotal role in myogenesis still is to be clarified, it is undeniable that vitamin D supplementation ameliorates proximal myopathy and muscle pain in patients with severe vitamin D deficiency [2, 117, 118]. Many reports on interventional studies are controversial; however vitamin D supplementation has been reported to improve musculoskeletal function in 12 weeks and reduce the risk of falls after 2 years in institutionalized subjects [5, 6, 119]. Based on their capability to balance immune system homeostasis, without being classical “immunosuppressants,” and target local inflammatory mediators at muscular level, VDR ligands appear to be optimal candidates as novel therapeutic agents for IMs. Furthermore, additional benefits of VDR agonists are related to their protective effects against bone-loss, infective pathogens, neoplasies [120–122], all side effects of immunosuppressive agents. Nevertheless, the use of VDR agonists in clinics is generally not pursued and is limited to calcipotriol, a drug applied for psoriasis topic treatment [123].

In fact, despite many advantages, the limit in therapeutic applications of vitamin D and vitamin D analogues undeniably relies on the systemic toxicity often associated with long-term intake: hypercalcemia is the main risk associated to the supraphysiological doses of vitamin D necessary to reach the low local effective concentration [124, 125]. Thus, the introduction of new molecules with immunosuppressive features without causing significant hypercalcemia has been strongly encouraged [126]. For this reason, drug development has been focusing on designing VDR agonists with a distinct separation between immunomodulatory and hypercalcemic potency. The selectivity is function of altered pharmacokinetics in comparison with the natural counterpart but, albeit quite many molecules eliciting much less calcemic effects have been developed, still discrepancies emerge between the therapeutic potential, as set in experimental work, and clinical data [127]. Those frustrating results may also depend on the lack of large and well designed trials.

Molecules with less or none hypercalcemic activity, as BXL-01-0029 or elocalcitol, could be suitable candidates—even as steroid-sparing agents—for inclusion in the therapeutic regimens for IMs. In particular, elocalcitol (150 µg/day P.O.) safety and tolerability in terms of calcemic effect has been proven in double-blind randomized study in 101 postmenopausal osteoporotic women, in placebo-controlled phase IIa (119 enrolled subjects) and follow-on phase IIb (514 patients) trial for the treatment of benign prostate hyperplasia (BPH) [128, 129]. Further studies bridging basic, clinic, and pharmacological researches seem mandatory and local administration strategies could be envisioned for IM treatment to overcome systemic drug intake.

As previously stated, inadequate response to therapy and, consequently, poor outcome are often encountered by IM patients. The major concern in clinics is the absence of internationally validated evaluation criteria to conduct randomized controlled trials [14, 15]: in fact, the few validated assessment tools available provide limited information helpful to patient management. To overcome this limit, multidisciplinary consortiums, such as the International Myositis Assessment and Clinical Studies Group (IMACS, http://www.niehs.nih.gov/research/resources/imacs/), or Paediatric Rheumatology International Trials Organization (PRINTO, https://www.printo.it/)—including rheumatologists, neurologists, physiatrists dermatologists, and other myositis experts—have been established to develop consensus and standards for the conduct and reporting of myositis [14]. Albeit recent advances in understanding the pathogenesis of myositis are unquestionably important, adequate multicentre trials with validated outcome measures represent a must to be pursued in order to define the best treatment for IMs and give clinical remission as a realistic objective to IM patients.
